# BK_Ca_ channel as a novel regulator of cellular DNA damage response in human bronchial epithelial cells in the presence of particulate matter

**DOI:** 10.1038/s41598-025-03824-9

**Published:** 2025-07-02

**Authors:** Kamila Maliszewska-Olejniczak, Agata Kustra, Wojciech Szymański, Adrianna Dąbrowska-Hulka, Monika Żochowska, Bogusz Kulawiak, Piotr Bednarczyk

**Affiliations:** 1 Department of Physics and Biophysics, Institute of Biology, Warsaw University of Life Sciences – SGGW, Warsaw, Poland; 2 Laboratory of Intracellular Ion Channels, Nencki Institute of Experimental Biology, Polish Academy of Sciences, Warsaw, Poland

**Keywords:** BK_Ca_ channel, DNA damage response, DNA-DSBs, DNA-SSBs, Apoptosis, PARP, DNA damage and repair, Molecular biology, Biochemistry, Ion channels

## Abstract

While particulate matter (PM) is a well-studied genotoxic environmental agent, our understanding of the molecular mechanisms through which PM triggers its harmful health consequences remains insufficient. The respiratory epithelium serves as the primary site for the deposition of PM, thereby acting as a protective barrier. These epithelial cells are characterized by the presence of notable potassium channels, which are critical for the regulation of the fluid layer. In human bronchial epithelial cells (HBE), the large-conductance Ca^2+^-regulated potassium (BK_Ca_) channels, localized to the apical site of the plasma membrane, are critical for the maintenance of proper airway surface liquid volume. In this work, we focused on the role of the BK_Ca_ channel and its potential role in DNA damage response (DDR) after PM exposure. The mechanisms of DDR have been extensively studied, however, the involvement of ion channels in this phenomenon is not known. Therefore, we used depleted for the BK_Ca_ channel HBE cells (HBE Δα BK_Ca_) as a physiological model. We demonstrated that exposure to standardized PM in HBE Δα BK_Ca_ cells induced reduced clone formation capabilities, an increase in ROS levels, PARP1-dependent apoptosis, cell cycle changes, and an increase in DNA double-strand breaks. A gene expression assessment by qPCR analysis revealed changes in expression levels of genes encoding proteins, especially from the DNA-single strand breaks repair pathway involved in oxidative DNA damage repair. Our findings imply that the absence of the BK_Ca_ channel might weaken the cellular response to DNA damage, potentially making cells more susceptible to PM-induced genomic instability. In conclusion, our research indicates the novel role of the BK_Ca_ channel in DDR for the first time.

**Supplementary Information:**

The online version contains supplementary material available at 10.1038/s41598-025-03824-9.

## Introduction

Air pollution poses a significant environmental health challenge that impacts populations worldwide. Recent clinical and epidemiological studies suggest that exposure to atmospheric particle matter (PM_2.5_) contributes to lung cancer, acute and chronic pulmonary inflammation, higher risk of stroke, neurodegenerative disorders, as well as sensory integration disorders^1–3^. The potential mechanisms by which PM influences the progression of these illnesses are associated with oxidative DNA damage, dysfunction of mitochondria, and inflammation at the level of individual cells, and with genotoxicity at the level of the genome^4,5^.

The respiratory epithelium serves as the primary site for the deposition of PM, thereby acting as a protective barrier against air pollutants. The integrity of the epithelial cells is preserved through apical junctional complexes, including tight and adherent junctions^6^. Human bronchial epithelial (HBE) cells are extensively studied for their barrier function in the airway epithelium, respiratory health effects, and ion transport mechanisms. These epithelial cells are characterized by the presence of channels, notably the sodium, chloride, and potassium channels, which are critical for the regulation of the liquid layer. In HBE cells, the large-conductance Ca^2+^-regulated potassium (BK_Ca_) channels, localized to the apical site of the plasma membrane, are critical for maintaining proper airway surface liquid volume^7^. However, various populations of BK_Ca_ channels are also present in cellular organelles such as mitochondria or nucleus^8,9^. Pore-forming α-subunit of BK_Ca_ channels from plasma membrane and organelles including mitochondria is encoded by a single *KCNMA1* gene^10–12^. Our recent study has highlighted the presence of a mitochondrial isoform of the BK_Ca_ channel, known as mitoBK_Ca_ in HBE cells^13^. We also found that PM harmfully impacts HBE cells at both cellular and mitochondrial levels, and mitoBK_Ca_ channels play a role in response to PM-induced damage^6^. Activation of mitoBK_Ca_ channels from various tissues induces mitochondrial depolarization, stimulates mitochondrial respiration, and regulates the synthesis of mitochondrial reactive oxygen species (ROS)^14^. Moreover, it has been shown that deleting the BK_Ca_/mitoBK_Ca_ channel pore-forming subunit increases mitochondrial and cellular ROS levels in glioma cells^15^. Furthermore, activation of mitoBK_Ca_ channels has been shown to induce cytoprotection against ischemia/reperfusion injury and the cytoprotective mechanism includes ROS attenuation induced by channel activation. The above research proposes that these channels may play a significant role in response to PM damage, which may be associated with the regulation of ROS.

The study of the role of BK_Ca_ channels in the physiology of the human bronchial epithelial (HBE) cells focuses explicitly on the mitochondrial (mitoBK_Ca_) and plasma membrane versions of these channels^13^. It has been shown that the same gene, *KCNMA1*, encodes both types of channels. Using CRISPR/Cas9 technology to disrupt this gene in HBE cells has led to the loss of both plasma membrane and mitochondrial BK_Ca_ channels, which was confirmed by electrophysiological experiments. It has also been shown that channel-deficient cells (HBE ΔαBK_Ca_) exhibited a significant decrease in transepithelial electrical resistance, which indicates a loss of tightness of the barrier created by these cells. A decrease in mitochondrial respiration has also been observed, indicating these organelles’ significant impairment. It concludes that the *KCNMA1* gene encodes both types of BK_Ca_ channels, which are crucial for maintaining the proper function of epithelial cells as a barrier^16^.

Although much research on PM exposure, especially regarding PM_10_ has been done in recent years, the mechanism of PM genotoxicity is poorly understood^17^. As two distinct genomes within one eukaryotic cell are present: nuclear and mitochondrial (mtDNA), prooxidative PM activity may affect both genomes inducing oxidatively damaged DNA and mitochondrial-nuclear cross-talk^18^. Ineffective or inaccurate repair of DNA damage triggers genomic instability, in the prolonged accumulation of mutations that are acknowledged to play a role in cancer development or death of the cell^19^. DNA repair capacity could be inhibited by some components of PM, especially metals (Cd^2+^, Ar^3+^, Co^2+^, Cr^6+^, Ni^2+^) which are known to inhibit the repair system machinery, and most of them are involved in carcinogenesis^20^. DNA damage triggers checkpoint mechanisms in mammalian cells, which lead to cell cycle arrest, allowing for DNA repair. In cases of severe damage, cell death, typically through apoptosis, may be induced^21^. The most genotoxic DNA lesions are DNA double-strand breaks (DNA-DSBs) which can be induced by different types of radiation^22^ or chemicals^23^. The model of DNA damage response and repair of DNA-DSBs is based on many studies connected to IR which is highly complex^22^. In mammalian cells, it has been developed two well-known mechanisms for DNA double-strand breaks (DSBs) repair: the non-homologous end-joining pathway (NHEJ) and homologous recombination repair (HR). Recent studies have demonstrated that exposure to PM not only leads to DSBs but also causes oxidative DNA damage, e.g. 8-oxo-deoxyguanosine (8-oxo-dG) which are the predominant adducts caused by PM exposure^24^. PM exposure leads to the phosphorylation of histone H2 AX (γH2 AX) at Ser-139 sites surrounding the damage^25^. The formation of γH2 AX at the sites of DNA-DSBs plays a crucial role in recruiting DNA repair proteins to facilitate the repair process^26^. γH2 AX serves as an indicator of DNA damage, particularly DSBs^27^. The phosphorylation of γH2 AX at DSBs spans up to 2 Mb flanking the breaks, enabling the recognition of γH2 AX foci formed at these sites through immunofluorescence. Base excision repair (BER) is the pathway for removing prevalent oxidative DNA damage, such as 8-oxo-dG. Another repair pathway that repairs mostly bulky DNA adducts is nucleotide excision repair (NER) which is inhibited by PM. This inhibition suppresses DNA repair and enhances replication errors^24^. Though, the molecular mechanisms underlying PM-induced DNA damage and repair response are not fully determined. It has been shown that nanoparticle-induced DNA damage could mimic irradiation-related carcinogenesis pathways^28^. If PM could mimic irradiation-related DNA damage response (DDR) pathways, the molecular mechanism of genotoxicity of PM could be associated with the role of ion channels like the transient receptor potential (TRP) channels as members of this family are involved in ionizing-radiation-induced cell death^29,30^. Exploring the interaction of these “gatekeepers” regulating ion flow across the cell membrane with DDR may reveal their impact on pivotal cellular pathways, including DNA repair, apoptosis, and cell cycle progression (reviewed in^31^.

In the current study, we aimed to elucidate the mechanisms by which HBE cells deficient in the α-subunit of the BK_Ca_ channel, function in response to PM-induced DNA damage. We examined the impact on clonogenic growth, ROS level changes, cell cycle alterations, and early apoptosis. In cases of severe or irreversible DNA damage, the arrested cell cycle ultimately results in the elimination of cells harboring DNA damage, employing apoptosis. Moreover, inducing permanent cell cycle arrest, known as senescence, is also a potential outcome^32^. Given that the analysis indicated cell death due to PM exposure, we evaluated apoptosis *via* the detection of PARP1 cleavage. Poly(ADP-ribose) polymerase 1 (PARP1) is an enzyme activated upon binding to DNA breaks, facilitating the poly(ADP-ribosyl)ation of different nuclear proteins, including itself, to attract DNA repair mechanisms to the damage sites^33^. It also acts as a target for caspases-3 and − 7 during caspase-dependent apoptosis. Cleavage of PARP1 by these caspases inactivates it, thus inhibiting DNA repair processes. This cleavage occurs close to the DNA-binding domain within a nuclear localization signal, producing fragments of 24 kDa and 89 kDa. Furthermore, we aimed to evaluate the alterations in gene expression levels encoding different proteins, representatives of each DNA repair pathway. Studying the involvement of BK_Ca_ channel in DNA damage may provide deeper insight into how these channels modulate cellular responses to genotoxic stress.

## Materials and methods

### PM sample

The particulate matter with a diameter of less than 4 μm (SRM-2786, NIST, Gaithersburg, MD, USA) was used in the current study^34^. To guarantee the consistency and repeatability of our biophysical and biochemical studies, we employed PM under the NIST standard. This particular PM was composed of atmospheric particulate material collected in Prague, Czech Republic. It was established at a cellular level that these samples represent reliable urban particles. Stock suspensions at 50 mg/ml were prepared in PBS and sonicated for 30 min to prevent clustering of the PM particles and stored at 4 °C. Experiments were performed using the freshly prepared stock solution to minimize variation in particulate matter composition. The suspensions were prepared to the final concentrations of 30, 50, and 100 µg/ml PM before direct application to the cell culture as performed in^17,20,35,36^.

### Cell culture and treatments

Human bronchial epithelial cells (16HBE14σ-, HBE) were obtained from Sigma-Aldrich, Inc. (St. Louis, MO, USA) and cultured according to the recommended conditions. The cells were cultured in a MEM medium with 10% FBS, penicillin, and streptomycin (10 mg/ml). Cells were cultured at 37 °C, 5% CO_2,_ and under 95% humidity. Growth media were changed according to the recommended conditions and cells were passaged at 70–80% confluence using trypsin-EDTA in PBS. Cells were visualized under CKX53 Olympus inverted microscope with Olympus Entry Cell Sense software v4.2. HBE Δα BK_Ca_ cell line was engineered using the precise CRISPR-Cas9 system according to Nature Protocols^37^ with gRNA designed and the physiology of these cells was described and published^16^. HBE cells were incubated with 30, 50, and 100 µg/ml concentrations of PM for 24 h directly in a cell culture medium. 50 µM etoposide (Sigma-Aldrich) with 2-hour treatment was used as a positive control for cell cycle shift and detection of DNA-DSBs.

### The clonogenic assay

The clonogenic assay is one of the available methods for assessing cell survival after cytotoxic treatment. It relies on the capacity of one cell to proliferate and form into a colony. The assay was determined as described previously with some modifications^35^. 500 cells were cultured on 6-well plates with 2 ml MEM medium (Sigma-Aldrich, St. Louis, MO, USA) and incubated for up to 9 days at 5% CO_2_ and 37 °C. After incubation, the HBE cell colonies were washed two times with 2 ml of PBS, then treated with 2 ml of 70% ethanol for 10 min and stained with 2 ml of Coomassie Brilliant Blue (Bio-Rad Laboratories, Inc.) staining solution (0.1% Coomassie Brilliant Blue R-250, 50% methanol, 10% glacial acetic acid, 40% distilled water) for 15 min, washed with 2 ml of Coomassie Blue Destaining solution (Bio-Rad Laboratories, Inc.), immersed in tap water and dried at room temperature. The number of colonies composed of over 50 cells was observed using a 10x objective under CKX53 Olympus inverted microscope and counted using countPHICS software^38^. Colony formation efficiency (plating efficiency, PE) was determined using the equation: (colony number/plated cell number) × 100%. The survival fraction (SF) was determined according to the equation: (PE of treated cells/PE of untreated cells) × 100% ^39^.

### Measurement of reactive oxygen species

Measurements of reactive oxygen species (ROS) were performed as previously described^6^. Upon reaching 90% confluence, cells were trypsinized and resuspended in a culture medium at a concentration of 500,000 cells/ml. Subsequently, 50,000 cells per well were seeded to a 96-well plate, following the incubation for 24 h. Next, the culture medium was aspirated, and the cells were washed with PBS. Following the wash, the cells were incubated for 30 min with 25 µM 2′,7′-Dichlorofluorescin diacetate (H2DCFDA; Invitrogen, Thermo Fisher Scientific, Waltham, MA, USA) for total ROS assessments. After the incubation period, 50 µg/ml PM was introduced and the cells were further incubated for 24 h, and fluorescence readings were obtained using a Fluoroskan Ascent (Thermo Fisher Scientific, Waltham, MA, USA) with excitation/emission at 485/538 nm.

### Flow cytometry analysis of cell cycle

For analyzing the cell cycle, 1 × 10^6^ HBE cells/sample after exposure to 50 µg/mL PM during 24 h under different experimental conditions (HBE wt, HBE Δα BK_Ca_, HBE wt + PM, HBE Δα BK_Ca_ + PM) were exposed to bromodeoxyuridine (BrdU, 10 µM final concentration) (51-2420 KC, BD Pharmingen) for 60 min using an Apoptosis, DNA Damage and Cell Proliferation Kit (BD Pharmingen), according to the manufacturer’s instructions. Then, the cells were trypsinized, washed with PBS, fixed and permeabilized with BD Cytofix/Cytoperm Fixation/Permeabilization Solution, BD Cytofix/Cytoperm Plus Permeabilization Buffer (51-2090 KE and 51-2356 KC, BD Pharmingen) and stained with PerCP-Cy5.5 mouse anti-BrdU antibody (51–9007682, BD Pharmingen) and DAPI (51–9007681, BD Pharmingen). After staining, samples were read using the BD LSR II flow cytometer and based on the BD FACSDiva Software v8.0.1. Each experiment was performed on different cell batches. The data were presented as an original multiparameter cell cycle analysis of the cell cycle phases (G0/G1, S, G2/M).

### Flow cytometry analysis of apoptosis

The number of HBE apoptotic cells after exposure to 50 µg/mL PM during 24 h was analyzed using an Apoptosis, DNA Damage, and Cell Proliferation Kit (BD Pharmingen), according to the manufacturer’s instructions. Up to 1 × 10^6^ cells under different experimental conditions (HBE wt, HBE Δα BK_Ca_, HBE wt + PM, HBE Δα BK_Ca_ + PM), were collected by trypsinization, washed in PBS, fixed and permeabilized with BD Cytofix/Cytoperm Fixation/Permeabilization Solution, BD Cytofix/Cytoperm Plus Permeabilization Buffer (51-2090 KE and 51-2356 KC, BD Pharmingen) and stained with PE Mouse Anti-Cleaved PARP (Asp214) Antibody (51–9007684) and DAPI (51–9007681, BD Pharmingen). The cleaved fragment of PARP (89 kDa) was used as an indicator of apoptosis. After incubation at 4 ^o^C, the HBE cells were analyzed using an LSR II flow cytometer (BD Biosciences LSR Fortessa, San Jose, CA, USA) based on BD FACSDiva Software v8.0.1. The experiments were performed using three independent biological repeats.

### Western blot analysis

Western blot analysis was performed as previously published^16^. Cells were harvested using trypsinization and lysed using cold RIPA buffer in a glass homogenizer (Wheaton Tight, USA). Protein concentrations were measured using the Bradford assay. 40 µg of protein was loaded on the gel. The proteins were resolved on a 10% Tris-Tricine SDS-PAGE gel and then transferred onto PVDF membranes (Bio-Rad Laboratories, Inc.). Blocking was performed with 10% non-fat milk in TBST buffer (50 mM TRIS, 150 mM NaCl, 0.2% Tween-20, pH 8.4) for 1 h at room temperature. Primary antibodies were diluted in TBST containing 5% non-fat milk. Anti-PARP1 (Anti-PARP Polyclonal Antibody, Thermo Fisher Scientific, USA, PA5-34803, 1:1000, 2 h) and β-actin antibodies (Anti-β Actin antibody, Abcam ab8227, 1:1000, 2 h) were used, followed by appropriate HRP-conjugated secondary antibodies (Anti-Rb, 1:5000) prepared in TBST with 5% non-fat milk. Detection of protein bands was achieved using ECL Prime Western Blotting Detection Reagent (Amersham, RPN 2236). Quantification of band intensity was performed using ImageJ software v1.54p.

### Flow cytometry analysis of DNA double-strand breaks

To assess the degree of DNA damage induced by exposure to PM, under different experimental conditions (HBE wt, HBE Δα BK_Ca_, HBE wt + PM, HBE Δα BK_Ca_ + PM) HBE cells were treated for 24 h with 50 µg/ml PM. Thereafter, cells were labeled with 10 µM of BrdU for 60 min., then 1 × 10^6^ of HBE cells were collected, and prepared for flow cytometry using PerCP-Cy5.5 mouse anti-BrdU antibody (51–9007682, BD Pharmingen) and Alexa Fluor 647 Mouse Anti-H2 AX (pS139) antibody (51–9007683, BD Pharmingen) for detection of DNA-DSBs from a kit dedicated for flow cytometry analysis and stained with DAPI (51–9007681, BD Pharmingen) (Apoptosis, DNA Damage and Cell Proliferation Kit, BD Pharmingen) according the manufacturer’s instructions. Samples were analyzed using an LSR II flow cytometer based on BD FACSDiva Software v8.0.1. The experiment was performed using three independent biological repeats. To calculate the relative amounts of γH2 AX, the ratio of fluorescent means (exposed HBE cells/unexposed HBE wt control) was used as previously described for flow cytometry analysis^40^.

### RNA isolation and cDNA synthesis

HBE cells were seeded in T-75 flasks and cultured to obtain a minimum of 1 × 10^6^ cells/flask. Then, for 24 h, cells were treated with 50 µg/ml PM and washed twice with PBS buffer. Total cellular RNA was isolated using a RNeasy Mini Kit (QIAGEN, TX, USA) according to the manufacturer’s instructions with DNase I (QIAGEN, TX, USA) treatment. RNA concentrations and purity were measured using µDrop Duo Plates in a Multiskan SkyHigh microplate reader (Thermo Fisher Scientific, DE, USA). The RNA (1 µg) was subjected to reverse transcription using an iScript cDNA Synthesis Kit (Bio-Rad Laboratories, Inc.) with RNase H^+^ MMLV reverse transcriptase according to the producer’s protocol.

### qPCR assay

The expression of selected 14 reference genes (listed in alphabetical order: *ACTB*,* B2M*,* G6PD*,* GAPDH*,* GUSB*,* HMBS*,* HPRT1*,* PGK1*,* RPL13 A*,* RPLP0*,* RPS18*,* TBP*,* TFRC*,* YWHAZ*) was screened with PrimePCR assay designed custom SYBR plate (Bio-Rad Laboratories, Inc.) according to the manufacturer’s protocol (Bio-Rad Laboratories, Inc.). Next, reference housekeeping genes with the highest level of stability between two HBE cell lines (HBE wt vs. HBE Δα BK_Ca_) were selected (*ACTB* encoding beta-actin, and *PGK1* encoding phosphoglycerate kinase 1) for the design of PrimePCR assay designed custom SYBR plates with 29 genes encoding proteins from DNA damage and DNA repair signaling pathways (listed in Supplementary Table S1). The reaction was performed using 1 µl of cDNA sample, iTaq Universal SYBR Green Supermix (Bio-Rad Laboratories, Inc.), and 20xPrimePCR assay primers dried in wells of 96-well plate according to the manufacturer’s protocol for custom plate (SYBR Green reaction setup). All qPCR experiments were performed using a CFX Opus 96 Real-Time PCR System (Bio-Rad Laboratories, Inc.). Gene expression data were analyzed using CFX Maestro Software v2.3 (Bio-Rad Laboratories, Inc.). Controls used in the study: PCR (tested performance of qPCR reaction with sample), RT (reverse transcription control assay tested performance of reverse transcription), RQ (RNA quality assay tested RNA integrity in sample). Fold change was calculated using the standard Eq. 2^-(ΔΔCt) as described previously^41^. The expression of each gene was calculated based on three biological replicates.

### Statistical analysis

All experiments were performed in three independent biological replicates to confirm reproducibility. Results were displayed as mean ± SD or mean ± SEM by Prism 4 (GraphPad Software Inc). One-way ANOVA was used to analyze experimental data. P-values were considered significant: *p ≤ 0.05, **p ≤ 0.01, ***p ≤ 0.001.

## Results

### Human bronchial epithelial cells are sensitive to PM exposure and cells lacking BK_***Ca***_ channel displayed reduced cell survival fractions

**Fig. 1:**
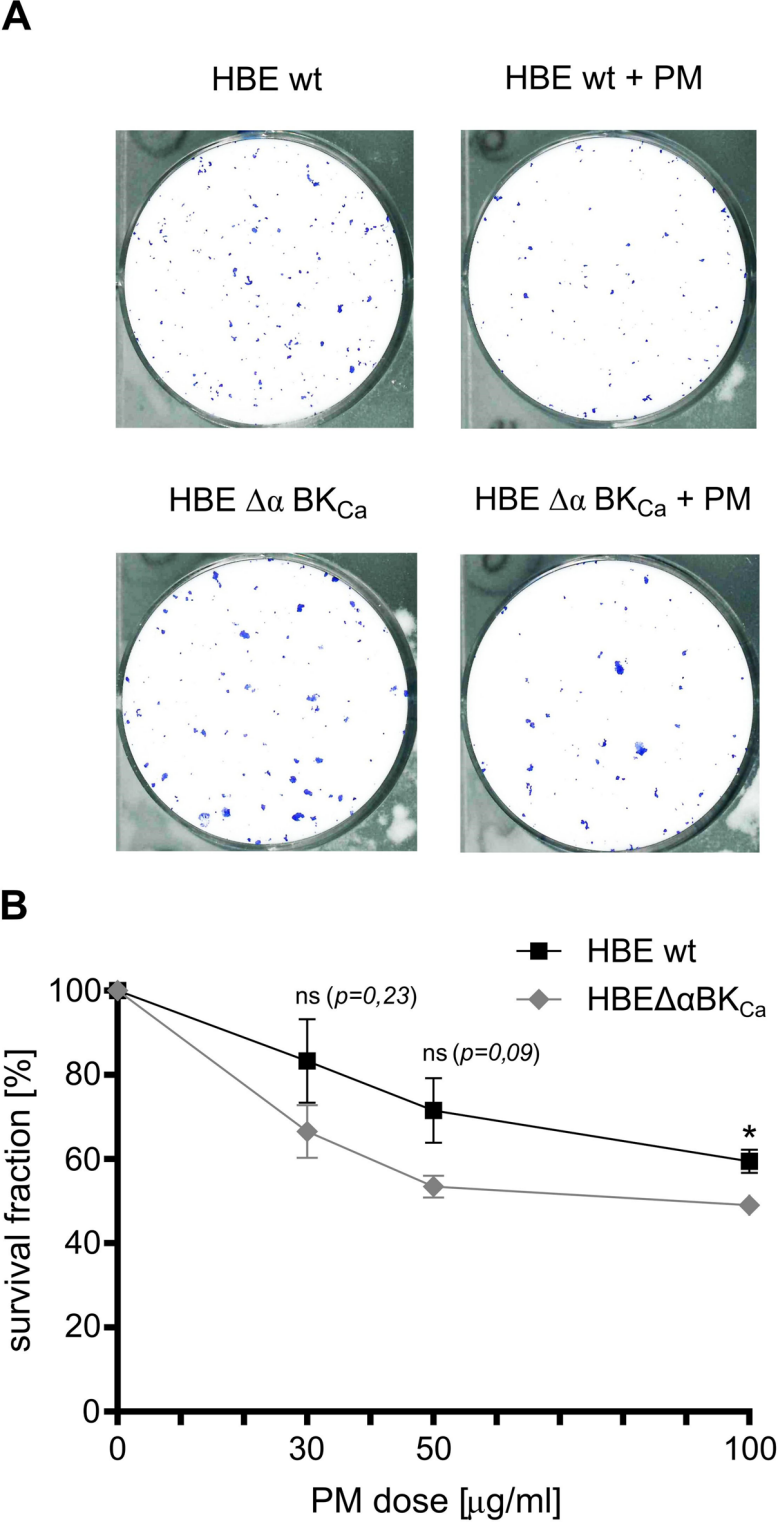
**Clonogenic survival of HBE wt cells** vs. **HBE Δα BK**_**Ca**_ **after cytotoxic 24-hour treatment with PM (0**,** 30**,** 50**,** and 100 µg/ml).** A: The representative images of colonies stained with Coomassie blue for nontreated and after 24-hour treatment of HBE cells with PM 50 µg/ml. B: The panel represents decreased colony survival fractions of HBE cells exposed to PM (SRM-2786) in comparison to the unexposed HBE cells. The colonies were analyzed after 9 days of incubation in 5% CO_2_ and 37 °C and counted using countPHICS software. Survival Fraction (SF) was considered using the formula: SF = (plating efficiency of tested cells/plating efficiency of control cells x 100%). Data were expressed as a percentage, the point bars represent the mean ± SEM (*n* = 3). One-way ANOVA was used to analyze experimental data. *P*-values were considered significant: *p ≤ 0.05, **p ≤ 0.01, ***p ≤ 0.001, ns – not significant.

In our study, we aim to understand the combined effects of PM on HBE cells and the influence of BK_Ca_ channels on these outcomes. To observe the anti-cell proliferation effect of BK_Ca_ knockout, firstly we adapted a colony formation assay to HBE cells. The colony formation assay is considered the gold standard and a fundamental test for assessing cell survival, commonly used to examine the effects of genotoxic agents^39^. Since the clonogenic assay is a method to assess cell reproductive death following exposure to ionizing radiation, we adapted and refined this methodology to evaluate the dose response to PM^42^. We established the cell survival fraction curve, which illustrates the correlation between the dose of a PM (0, 30, 50, and 100 µg/ml) and the number of surviving colonies. In this set of experiments we compared the effects of PMs on HBE wt cells vs. HBE Δα BK_Ca_ (Fig. 1). At a concentration of 50 µg/ml PM, the cell survival rate in HBE Δα BK_Ca_ cells was 53.42% ± 3.49% in comparison with HBE WT cells (71.55% ± 9.67%) (ns, *p* = 0.09), indicating a higher sensitivity of the HBE Δα BK_Ca_ line to PM-induced effects. For HBE WT cells, the cell survival fraction at 100 µg/ml PM was 59.47% ± 3.67%, while for HBE Δα BK_Ca_ cells it was 49.01% ± 1.27% (*p ≤ 0.05). Based on these results, the concentration of 50 µg/ml was selected for further experiments in order to avoid excessive cytotoxicity and to allow the assessment of moderate, non-lethal, and potentially indirect cellular effects.

### PM exposure increased ROS levels in both WT cells and cells lacking BK_Ca_ channel

**Fig. 2:**
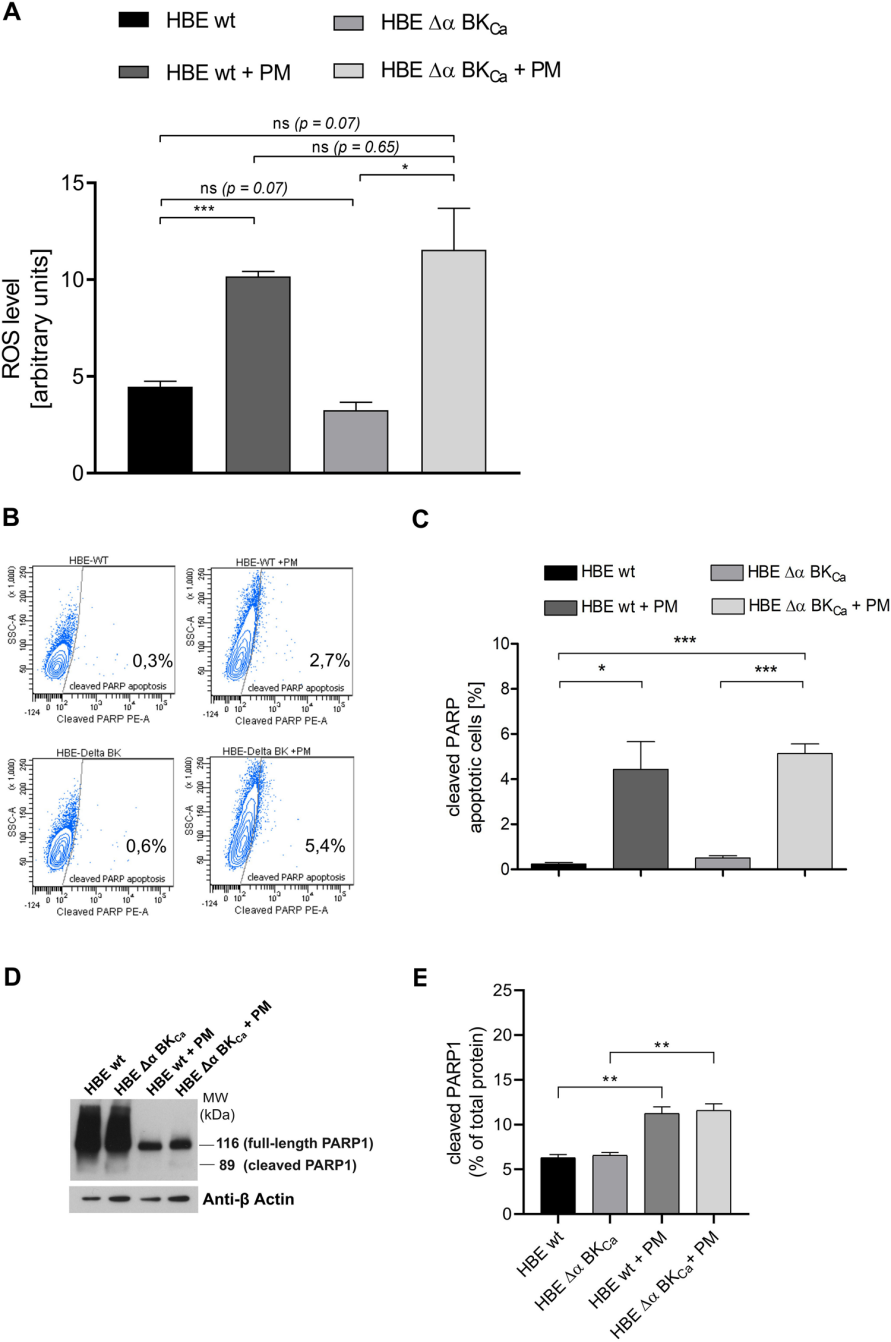
**The influence of PM exposure (50 µg/ml**,** 24 h) on HBE wt cells** vs. **HBE Δα BK**_**Ca**_ **on intracellular ROS level (in arbitrary units) and cleaved PARP apoptosis.** A: Data represent total ROS measurements and are expressed as mean ± SD (*n* = 3) for ROS level analysis (H2DCFDA probe). B: Representative images of flow cytometry analysis PARP1 cleavage. The fragment of cleaved-PARP1 (89 kDa) was used as a marker of apoptosis (PE mouse anti-cleaved PARP (Asp214) antibody). C: The data were expressed as a percentage, the point bars represent the mean ± SEM (*n* = 3) for apoptosis. D: Western blot analysis of PARP1 protein expression levels (left panel). The anti-PARP1 antibody detects both the full-length PARP1 protein and its 89-kDa cleaved fragment. β-actin served as the loading control. E: Quantitative analysis (optical density) (right panel). The bar graphs show the levels of cleaved PARP1, based on pooled densitometric analyses (mean ± SEM, *n* = 3). Results are expressed as the ratio of cleaved protein to total protein, normalized accordingly. One-way ANOVA was used to analyze experimental data. *P*-values were considered significant: *p ≤ 0.05, **p ≤ 0.01, ***p ≤ 0.001, ns – not significant.

Exposure to 50 µg/ml PM for 24 h significantly increased intracellular ROS levels in both HBE WT and HBE Δα BK_Ca_ cells compared to non-treated controls, although no significant difference was observed between the two cell lines (Fig. 2A). The intracellular ROS level rates for HBE wt and HBE Δα BK_Ca_ cells were 10.17% ± 0.44, and 11.54% ± 4.88 respectively after the PM exposure. Although HBE ΔαBK_Ca_ cells exhibit a trend toward elevated ROS levels upon PM exposure, this difference does not reach statistical significance. However, there is increased signal gain (WT/WT + PM vs. Δα BK_Ca_/Δα BK_Ca_+PM). This suggests that while BK_Ca_ may have a modest influence on ROS accumulation, the current results do not definitively establish a direct role for BK_Ca_ in ROS control. Additional future studies are necessary to clarify BK_Ca_’s contribution under varying experimental conditions. The collection of higher levels of ROS will intensify oxidative stress, leading to oxidative DNA damage, proteins, and lipids^43^. Therefore, we planned to investigate the role of the BK_Ca_ channel in the cellular response to PM-induced DNA damage (DNA damage-induced cell cycle changes, apoptosis, DNA damage and expression of repair genes).

### Particulate matter induced early apoptosis via caspase-3 cleavage of PARP-1 in HBE cell lines

PARP1 undergoes proteolytic cleavage by caspases-3 and − 7, resulting in two fragments: a 24-kDa N-terminal and an 89-kDa C-terminal fragment^33^. The cleaved-PARP fragment (89 kDa) was used in our studies as an apoptosis marker using PE Anti-Cleaved PARP (Asp214) antibody for cytometry analysis (Fig. 2B, C). Our results demonstrated that both analyzed cell lines displayed increased PM-induced early apoptosis *via* caspase-3 cleavage of PARP1. After treatment with 50 µg/ml for 24 h, the HBE wt and HBE Δα BK_Ca_ cells showed increased apoptosis rates of 4.43% ± 1.58 and 5.13% ± 0.56 respectively and the differences were statistically significant. Western blot analysis using an anti-PARP1 antibody—capable of detecting both the full-length protein and its 89-kDa cleaved form—revealed the presence of the 89-kDa fragment following 24-hour exposure to 50 µg/ml PM (Fig. 2D and Figure S3). Densitometric quantification showed that the cleaved form of PARP1 accounted for approximately 11.25% ± 0.89 in HBE wt + PM and 11.59% ± 0.88 in HBE Δα BK_Ca_ + PM cells, compared to 6.3% ± 0.42 and 6.56% ± 0.42 in untreated HBE wt and Δα BK_Ca_ controls, respectively (Fig. 2E). These results indicate that exposure to 50 µg/ml PM induces PARP1 cleavage in both HBE wt and HBE Δα BK_Ca_ cells, suggesting activation of the early apoptotic pathway. The increased proportion of cleaved PARP1 compared to untreated controls supports the involvement of caspase-dependent mechanisms in response to PM exposure. These findings are consistent with and further support the flow cytometry data indicating elevated levels of apoptosis under the same conditions.

### Cell cycle dynamics in HBE cells: examining the influence of BK_***Ca***_ deletion and after PM exposure

**Fig. 3:**
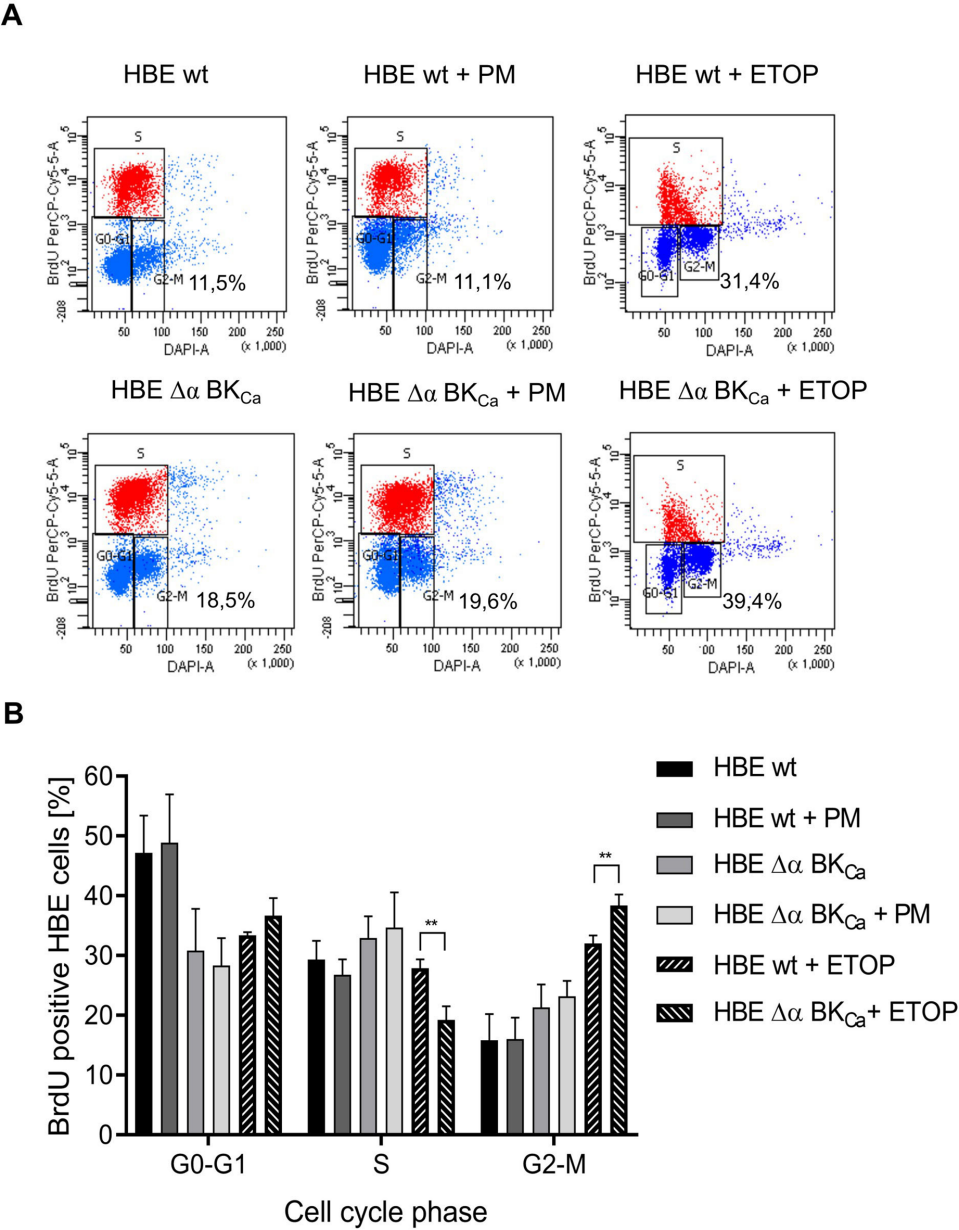
**Flow cytometry identification of cell cycle changes with anti-BrdU antibody in HBE wt and HBE Δα BK**_**Ca**_ **cells after PM exposure (50 µg/ml**,** 24 h).** The cells were labeled with 10 µM BrdU for 1 h and stained with BrdU PerCP-Cy 5.5 antibody. Flow cytometry representative images (A panel) of DAPI versus BrdU PerCP-Cy 5.5 staining profile (% of HBE cells in G2/M phase) and relative quantification (B panel). Cells were properly gated: G0/G1 phase, S phase and G2/M phase. 50 µM etoposide (ETOP) was used as a positive control (2-hour incubation). Data were demonstrated as percentages of HBE cells and the bars correspond to the mean ± SEM (*n* = 3). One-way ANOVA was used to analyze experimental data. *P*-values were considered significant: *p ≤ 0.05, **p ≤ 0.01, ***p ≤ 0.001, ns – not significant.

Immunofluorescent staining for HBE cells that have integrated Bromodeoxyuridine (BrdU), combined with flow cytometric analysis, provides a detailed method for identifying the occurrence and characteristics of cells that have undergone DNA synthesis. To further investigate the effect of PM on the proliferation of HBE cells, we examined the cell cycle after a 24-hour treatment with 50 µg/ml of PM (Fig. 3A and B). Incorporation of BrdU indicated that the percentage of cells in the G2/M phase was around 15.8 ± 5.9% in HBE wt cells and 16.0 ± 4.7% after PM exposure (Fig. 3B). HBE Δα BK_Ca_ cells showed increased rates of 21.3 ± 5.1% and 23.2 ± 3.3% after PM exposure. The halt of the cell cycle in the G2/M phase may imply that intracellular DNA damage is challenging to repair^44^. Upon detecting changes in DNA structure, an effective response to DNA damage involves halting the cell cycle and initiating relevant repair processes through the initiation of diverse DNA-repair machinery^45^. In cases of severe or irreversible DNA damage, the arrested cell cycle ultimately results in the elimination of cells harboring such damage, employing apoptosis. Moreover, inducing permanent cell cycle arrest, related to cellular senescence, is also a potential outcome. HBE ΔBK_Ca_ cells exhibit a distinct cell cycle distribution even in the absence of PM exposure, suggesting that the deletion of the BK_Ca_ channel may affect cell cycle dynamics. However, the lack of statistically significant differences across conditions and the variability observed between biological replicates suggest that these changes might not be solely attributable to BK_Ca_ deletion. Instead, they could be linked to broader cellular stress responses or genomic stability mechanisms. Interestingly, following treatment with the positive control etoposide, cells lacking the BK_Ca_ channel exhibited the most pronounced accumulation in the G2/M phase (38.37 ± 1.44%). This may suggest a potential involvement of the BK_Ca_ channel in the regulation of cell cycle progression and DNA damage response mechanisms. To further investigate the potential consequences of these cell cycle alterations, we extended our study to assess DNA damage and DDR gene expression.

### Cells lacking BK_***Ca***_ channel displayed increased DNA-double strand breaks via histone γH2 AX phosphorylation induced by PM

**Fig. 4:**
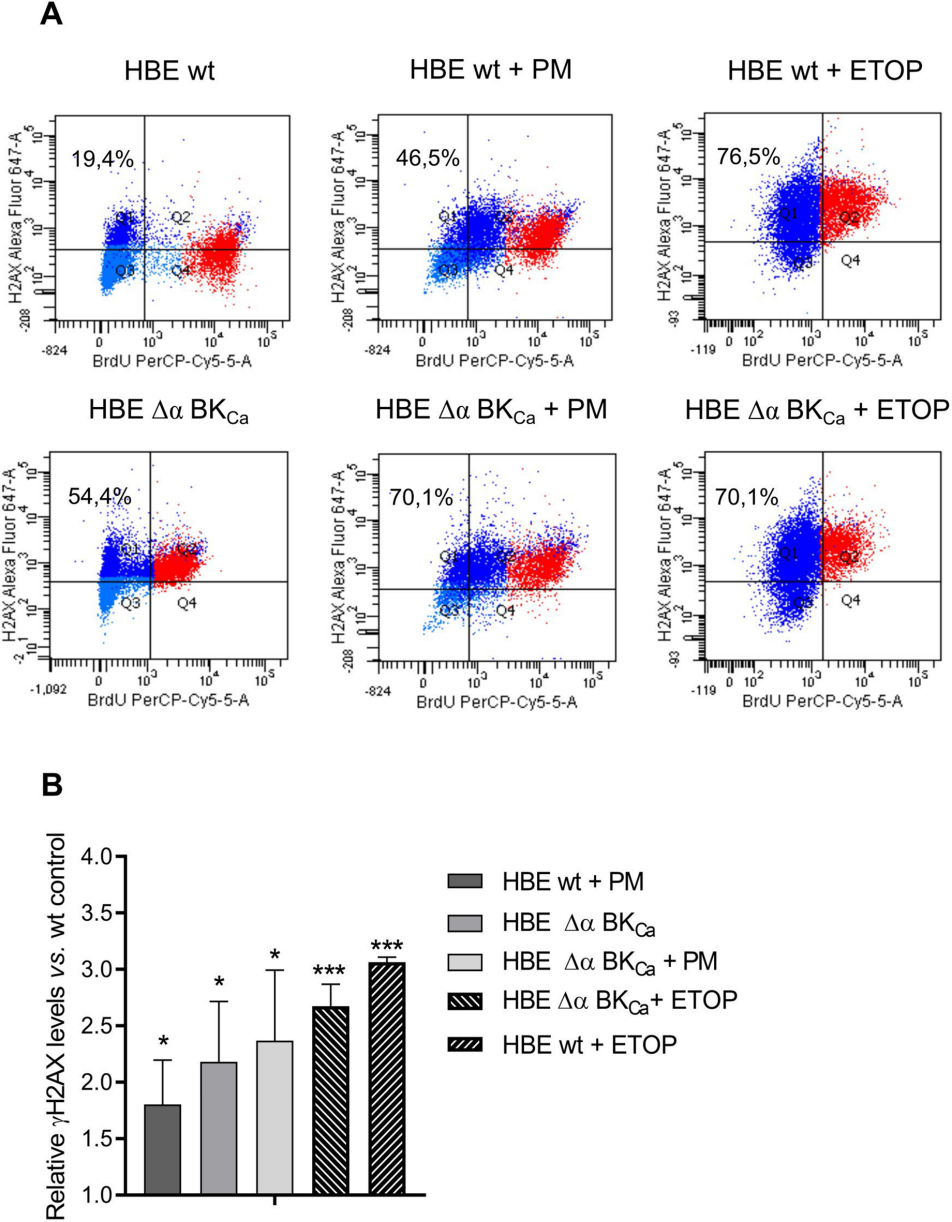
**Identification of DNA damage in HBE cells after PM exposure (50 µg/ml**,** 24 h) with antibody to histone H2 AX (pS139).** A: Flow cytometry representative images of BrdU PerCP-Cy 5.5 versus H2 AX (pS139) Alexa Fluor 647 profile (% indicating the proportion of DNA double-stranded breaks in presented experiment). BrdU-positive cells are color-gated red and DNA damage is dark blue. B: Relative quantification from three independent experiments. Levels of γH2 AX were normalized to untreated HBE wt control set to unity and the bars represent the mean ± SEM (*n* = 3). 50 µM etoposide (ETOP) was used as a positive control (2-hour incubation). One-way ANOVA was used to analyze experimental data. *P*-values were considered significant: *p ≤ 0.05, **p ≤ 0.01, ***p ≤ 0.001, ns – not significant.

Flow cytometry analysis of γH2 AX allows for the quantification of DNA damage extent in individual cells and the association of damage with DNA content^44^. Therefore, the γH2 AX served in our studies as a marker of DNA-DSBs using Alexa Fluor 647 Mouse Anti-H2 AX (pS139) Antibody. We conducted flow cytometry analysis of γH2 AX, enabling a comprehensive assessment of DNA damage extent in individual cells to associate the extent of damage with DNA content. We demonstrated after exposure to 50 µg/ml PM (24 h) an increase in DNA-DSBs in HBE Δα BK_Ca_ cells in comparison to the unexposed HBE wt cells (Fig. 4). Moreover, in cells lacking a BK_Ca_ channel, we have also observed an increased level of γH2 AX, however lower but comparable with those treated with PM. Measurement of γH2 AX distribution in HBE cells exposed to PM revealed elevated ratios of DNA-DSBs in comparison to the nonexposed HBE wt cells: 1.8 ± 0.49 (HBE wt + PM), 2.18 ± 0.68 (HBE Δα BK_Ca_) and 2.37 ± 0.83 (HBE Δα BK_Ca_ + PM) (Fig. 4B). We demonstrated an increase of DNA double-strand breaks occurrence in HBE cells depleted for the BK_Ca_ channel indicating for the first time the involvement of this channel in the DNA damage response in a highly interconnected manner. The absence of BK_Ca_ leads to an increase in DNA-DSBs compared to HBE wt cells, which could predispose the cells to greater sensitivity to PM-induced DNA damage. Our findings propose that the BK_Ca_ channel may play a significant role in the molecular mechanisms of DNA damage response and repair following exposure to particulate matter.

### Cells lacking BK_***Ca***_ channel displayed altered DDR gene expression levels as measured by qPCR analysis after exposure of HBE cells to PM

To check that the BK_Ca_ channel can be a critical component of the DNA damage response machinery, we examined changes in gene expression levels in HBE cells after exposure to 50 µg/ml PM for 24 h. Total RNA from nonexposed and PM-exposed HBE cells was used to perform qPCR arrays in 96-well designed plates. We utilized the commercially provided custom PCR arrays, which have been designed to evaluate the expression of 29 selected genes (Table S1) involved in DNA damage signaling. These arrays allow fast and reliable analysis of gene expression related to specific pathways, positioning them as an essential initial step in identifying genes with altered expression under certain conditions. They serve as an alternative to the more elaborate and expensive microarray analyses.

Firstly, we identified reference genes with the most stable gene expression for HBE wt and HBE Δα BK_Ca_ cells among tested 14 reference genes (*ACTB*,* B2M*,* G6PD*,* GAPDH*,* GUSB*,* HMBS*,* HPRT1*,* PGK1*,* RPL13 A*,* RPLP0*,* RPS18*,* TBP*,* TFRC*,* YWHAZ*). This group of all reference genes is stable and represents minimal variations across the tested cells. However, *ACTB* (encoding beta-actin) and *PGK1* (encoding phosphoglycerate kinase 1) reference genes were chosen for further studies according to their highest gene expression level stability plot (Fig. S1).

**Fig. 5:**
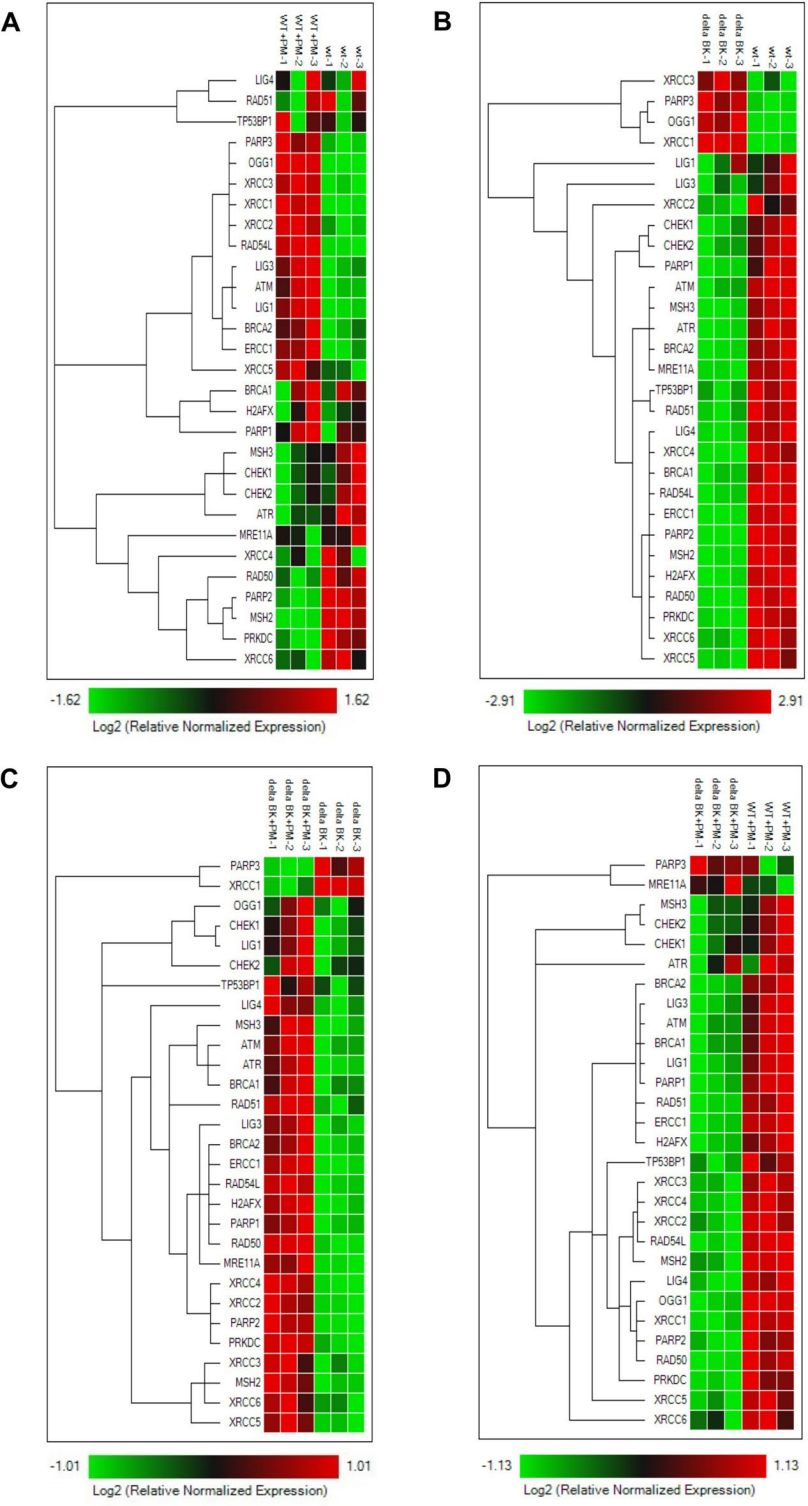
**Cluster heatmap analysis of the normalized expression of DNA-damage signaling genes after PM exposure (50 µg/ml**,** 24 h).** A: HBE wt vs. wt + PM; B: Δα BK_Ca_ vs. wt; C: Δα BK_Ca_ vs. Δα BK_Ca_ + PM; D: wt + PM vs. Δα BK_Ca_ + PM. The red color characterizes a relatively high level of gene expression whereas the green color indicates a low level. The data were analyzed (*n* = 3) and clustered by targets using the Reference Gene Selection Tool from CFX Maestro Software v2.3 (Bio-Rad Laboratories, Inc.).

**Fig. 6:**
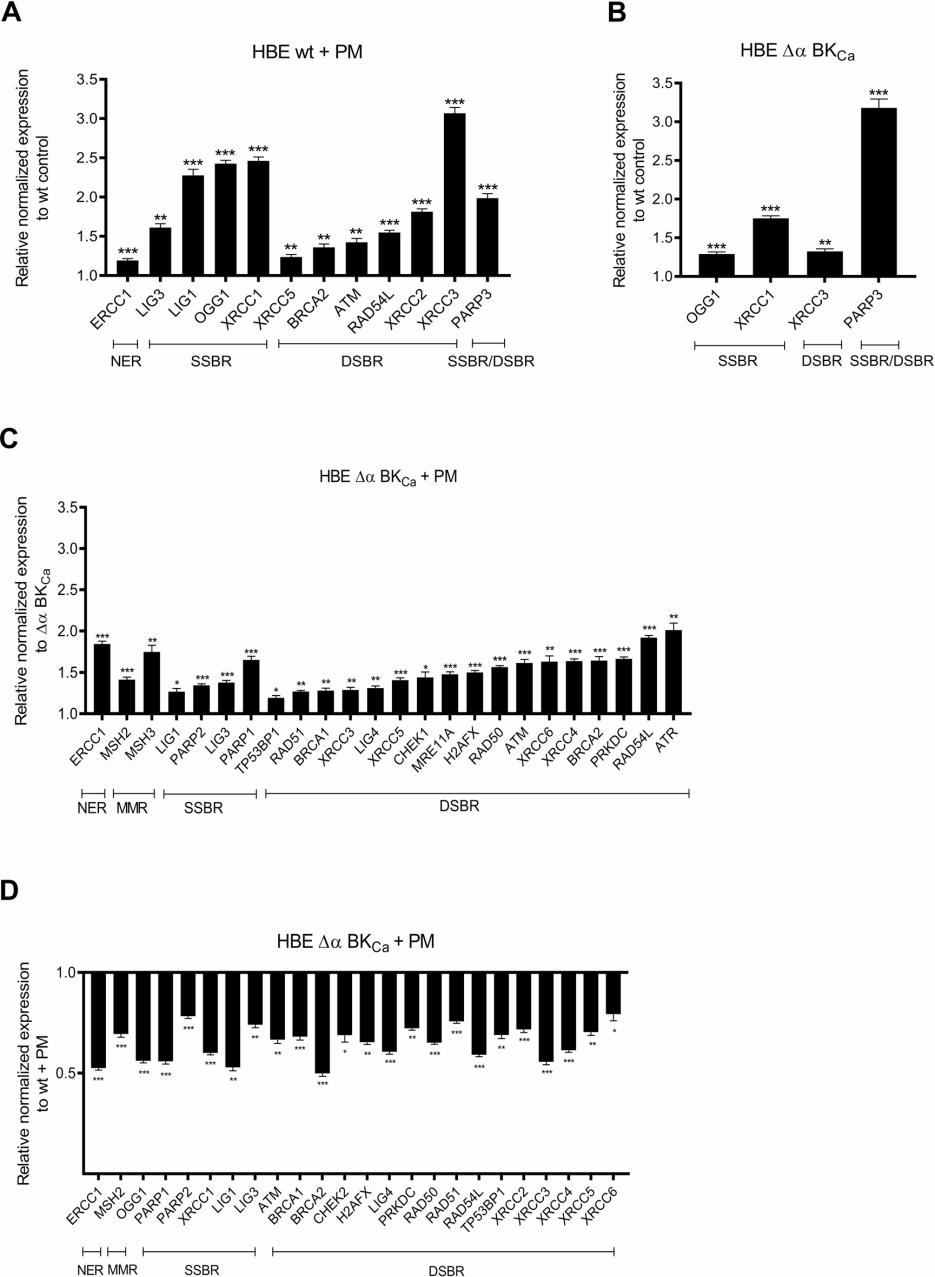
**Relative to control the normalized expression of DNA-damage response and repair genes after PM exposure (50 µg/ml**,** 24 h).** A: HBE wt vs. wt + PM; B: Δα BK_Ca_ vs. wt; C:. Δα BK_Ca_ vs. Δα BK_Ca_ + PM; D: wt + PM vs. Δα BK_Ca_ + PM. NER: the nucleotide excision repair, MMR: DNA mismatch repair, SSBR: single-strand breaks repair, DSBR: double-strand breaks repair. Bar plot of the relative expression of all genes calculated *via* the ΔΔCt method. The bars correspond to the mean ± SEM (*n* = 3). One-way ANOVA was used to analyze experimental data. *P*-values were considered significant: *p ≤ 0.05, **p ≤ 0.01, ***p ≤ 0.001, ns – not significant.

Next, we examined changes in gene expression levels in 4 groups: A: HBE wt cells vs. wt + PM; B: Δα BK_Ca_ vs. wt; C: Δα BK_Ca_ vs. Δα BK_Ca_ + PM; D: wt + PM vs. Δα BK_Ca_ + PM; (Figs. 5 and 6).

The first group (A) was selected to demonstrate changes in gene expression levels in control HBE cells after PM exposure, as PM induces DNA damage, and therefore may contribute to changes in gene expression, so we planned to test which genes are upregulated in our HBE cell model. In the HBE wt cells, higher levels in the expression of genes belonging to DNA-damage signaling pathways were noted in response to PM exposure (Figs. 5A and 6A). The most substantial alterations from the control levels were detected for the upregulation of 12 genes among 29 tested (*PARP3*,* OGG1*,* XRCC3*,* XRCC1*,* XRCC2*,* RAD54L*,* LIG3*,* ATM*,* LIG1*,* BRCA2*,* ERCC1*,* XRCC5*). Among this group, 6 genes were highly expressed: *LIG1*,* OGG1*,* PARP3*,* XRCC1*,* XRCC2*, and *XRCC3* (Figs. 5A and 6A and S2). *LIG1*,* OGG1*, and *XRCC1* are involved in the SSBR (single-strand break repair) pathway, and *XRCC2* and *XRCC3* in the DSBR (double-strand break repair) pathway. Interestingly, PARP3 (poly(ADP-ribose) polymerase 3) is a multifunctional member of the PARP family that catalyzes mono-ADP-ribosylation (MARylation) of itself, various protein substrates, and DNA termini at both single- and double-strand breaks^46^. Initially identified as a regulator of classical non-homologous end joining (C-NHEJ), PARP3 has been implicated in the DSBR^47^. However, accumulating evidence highlights its role in SSBR as well, suggesting its broader involvement in DNA damage response pathways. Therefore, in our analyses, we classified this protein as involved in both SSBR/DSBR pathways. Beyond DNA repair, PARP3 modulates chromatin structure and gene expression by interacting with chromatin regulators. Its catalytic activity opposes G9a-mediated repression of adhesion and hypoxia-responsive genes^46^. Moreover, PARP3 has been associated with promoting chromosomal rearrangements and enhancing breast cancer aggressiveness.

The second group (B) was identified to verify differences in expression levels of genes involved in DDR between non-treated cells: HBE Δα BK_Ca_ cells vs. HBE wt cells. The highest differences were observed for gene *PARP3* belonging to SSBR/DSBR pathways, *XRCC1* and *OGG1* belonging to the SSBR pathway, and *XRCC3* belonging to the DSBR pathway (Figs. 5B and 6B and S2). *XRCC3* encodes a member of the RecA/Rad51-related family, contributing to DSBR-HR repair and the maintenance of chromosomal stability^48^. *XRCC1* is required to maintain genome stability *via* its participation in DNA repair^49^. The main functions of *XRCC1* and 8-oxoG-glycosylase 1 (*OGG1*) are related to their role in the SSBR and BER pathways, which possess several enzymatic stages in common.

The third group (C) was selected to demonstrate changes in gene expression levels in HBE Δα BK_Ca_ cells after PM exposure to see if the lack of a BK_Ca_ channel induces altered gene expression levels in DDR signaling. In this group, 24 genes were significantly upregulated, where only 2 genes were downregulated (*PARP3* and *XRCC1*) (Figs. 5C and 6C and S2). Lack of BK_Ca_ channel induced after PM exposure activation of genes encoding almost all DDR pathways. The obtained results strongly suggest implications for the role of the BK_Ca_ channel in DNA repair and maintenance.

The fourth group (D) was selected to demonstrate DNA-damage signaling in HBE wt cells versus Δα BK_Ca_ cells, both after PM exposure. In this group, only 2 genes were upregulated among 29 tested genes (*PARP3* and *MRE11 A*) (Fig. 5D, and S2), though without statistical significance. MRE11 is a nuclease that functions as part of the MRN complex, which consists of MRE11, RAD50, and NBS1^50^. It exhibits both endonuclease and exonuclease activities, enabling short-range 3’ to 5’ resection of DNA ends and processing of blocked DNA termini. These functions are essential for the initiation of double-strand break repair. Therefore, MRE11 is classified as a key component of the DSBR pathway. Notably, the 24 of tested genes (*ERCC1*, *MSH2*, *OGG1*, *PARP1*, *PARP2*, *XRCC1*, *LIG1*, *LIG3*, *ATM*, *BRCA1*, *BRCA2*, *CHEK2*, *H2 AFX*, *LIG4*, *PRKDC*, *RAD50*, *RAD51*, *RAD54L*, *TP53BP1*, *XRCC2*, *XRCC3*, *XRCC4*, *XRCC5*, *XRCC6*) in the Δα BK_Ca_ cell line were significantly downregulated following PM exposure (Figs. 5D and 6D) in comparison with wt cells, suggesting a potential impairment in the DNA damage response. This downregulation may indicate a reduced capacity for DNA repair, particularly in pathways responsible for maintaining genomic stability under environmental stress conditions. These findings imply that the absence of the BK_Ca_ channel might weaken the cellular response to DNA damage, potentially making cells more susceptible to PM-induced genomic instability.

## Discussion

Eukaryotic cells have developed an extensive array of DNA repair mechanisms to cope with the diverse types of DNA damage originating from both external and internal sources^51^. Impairments in these repair mechanisms result in enhanced or early aging and heighten the risk of developing cancer. Genomic instability, a hallmark feature of cancer, is linked to the buildup of DNA damage. Focused cancer therapies that target the DNA damage response present an opportunity to expand the therapeutic range. When cells are exposed to agents that damage DNA, the DDR signaling becomes crucial in determining cellular sensitivity. Recent research indicates that the BK_Ca_ channel emerges as a novel target for anticancer therapy, directing attention toward possible innovative therapeutic strategies^52–54^. Our study highlights the significant role of large-conductance Ca^2+^-regulated potassium (BK_Ca_) channels in the DNA damage response induced by particulate matter. This finding is critical as previous research predominantly focused on the roles of cytoplasmic and nuclear proteins in DDR, with less attention given to ion channels like BK_Ca_. The implication of BK_Ca_ channels in DDR, especially in HBE cells, suggests a novel pathway through which PM exerts its genotoxic effects.

The observed reduction in colony formation capabilities upon PM exposure aligns with existing literature that demonstrates the genotoxic nature of PM^1,4,17,18,25,36,55–63^. Moreover, our results may suggest that HBE Δα BK_Ca_ cells display increased PM-induced sensitivity. While the BK_Ca_ channel may have a modest influence on ROS accumulation, the current results do not definitively establish a direct role for BK_Ca_ in ROS control. Additional future studies are necessary to clarify BK_Ca_’s contribution under varying experimental conditions. Moreover, exposure to 50 µg/ml PM induced PARP1 cleavage in both HBE wt and HBE ΔαBK_a_ cells, indicating early, caspase-dependent apoptosis consistent with flow cytometry results.

The observed increase in DNA double-strand breaks and G2/M DNA damage checkpoint cell cycle arrest may suggest a heightened level of DNA damage, potentially aligning with mechanisms previously noted in PM_2.5_-induced cytotoxicity, though additional data would strengthen this hypothesis^63^. Our study suggests a potential association between these effects and the depletion of BK_Ca_ channels, although further investigations are needed to confirm this link. However, we demonstrated that the absence of BK_Ca_ leads to an increase in DNA-DSBs compared to HBE wt cells, which could predispose the cells to greater sensitivity to PM-induced DNA damage. The upregulation of genes involved in the DNA single-strand breaks repair (SSBR) and DNA double-strand breaks repair (DSBR) pathway in HBE Δα BK_Ca_ cells is a significant finding. This observation aligns with studies that have demonstrated the upregulation of DDR genes in response to environmental stressors^25^. The specific increase in expression levels of genes like *PARP3*,* OGG1*,* XRCC1*, and *XRCC3* in our study points towards an intricate regulatory network being activated in response to PM exposure and BK_Ca_ channel depletion. PARP3, belonging to the PARP family, facilitates post-translational modifications of proteins to regulate various cellular processes, such as genome stability, transcription, cellular metabolic processes, and apoptosis. Recent developments in the specific functions of PARP3 in tumor aggressiveness were described^64^. Interestingly, PARP3 has been associated with promoting chromosomal rearrangements, limiting G4 DNA structures, and enhancing breast cancer aggressiveness, pointing to its selective inhibition as a potential therapeutic strategy in oncology^46^. *OGG1*,* XRCC1*, and *XRCC3* participate in the repair of oxidized bases, SSBs, and DSBs. Genetic alterations in these genes have been linked to a heightened susceptibility to cancer^65^. The upregulation of *XRCC3*,* XRCC1*, and *XRCC2* indicates an enhanced activation of homologous recombination and base excision repair pathways. *OGG1* upregulation suggests an increased response to oxidative stress. Since OGG1 repairs DNA damage from reactive oxygen species, it’s possible that the BK_Ca_ channel could influence how cells handle oxidative stress caused by particulate matter exposure. This connection needs further study to better understand its implications. On another hand, calcium plays a crucial role in genotoxic stress due to hyperactivation of PARP-1 following alterations in cellular metabolism and DNA repair, which are induced by ROS-caused DNA damage^66^. The increased expression of *LIG1* and *LIG3* could point to a higher need for DNA ligases, which help repair DNA by sealing breaks and gaps. This might be a way cells compensate for DNA damage when BK_Ca_ channels are disrupted. Our analysis of gene expression between both cell lines post-PM exposure reveals that, notably, a majority of genes were downregulated in the Δα BK_Ca_ cell line compared to the wild-type cells. This suggests a potential impairment in the DNA damage response mechanism. Such downregulation might indicate a diminished capacity for DNA repair, especially in pathways crucial for maintaining genomic stability under environmental stress. These observations imply that the absence of the BK_Ca_ channel could weaken the cellular defenses against DNA damage, potentially rendering the cells more vulnerable to PM-induced genomic instability. The BK_Ca_ channel might play a role in maintaining cellular homeostasis by contributing to DNA repair processes. This indicates the novel, cytoprotective role of the BK_Ca_ channel in this process.

The interaction between BK_Ca_ channels and PM exposure may be crucial for maintaining cellular homeostasis. It has been reported that BK_Ca_ channels modulate ROS production, gene expression, and cell cycle progression, thereby maintaining cellular integrity^9,12,67^. The gene expression is controlled by the nuclear BK_Ca_ channel located within the nucleus of hippocampal neurons through the modulation of nuclear calcium (Ca^2+^) signaling^9^. The significance of reactive oxygen species production was demonstrated as a crucial mitochondrial function implicated in cellular adaptation and resistance to stress^68^. Mitochondrial ROS were demonstrated to be crucial in promoting the progression of the cell cycle^69^. There is a potential overlap between the pathways affected by BK_Ca_ channel activity and those altered by PM exposure, suggesting a converging mechanism of action. Understanding the interplay between BK_Ca_ channels and PM exposure could provide insights into the mechanisms of environmental pollutant-induced diseases. Given the role of BK_Ca_ channels in modulating DDR in HBE cells exposed to PM, there is potential for these channels to be explored as therapeutic targets. This aligns with emerging research suggesting ion channels as novel targets in disease treatment, particularly within the framework of environmental toxin-induced damage^31^. Future research should emphasize elucidating the detailed mechanisms by which BK_Ca_ channels influence DDR pathways. Investigating the interactions between BK_Ca_ channels and key DDR proteins could provide deeper insights into how these channels modulate cellular responses to genotoxic stress. Additionally, exploring the effects of BK_Ca_ channel modulation in in vivo models of PM exposure would be valuable in confirming the physiological relevance of our findings.

Our study provides new insights into the molecular mechanisms of PM-induced DNA damage, particularly highlighting the novel role of BK_Ca_ channels in DDR, showing for the first time evidence at the molecular level in DDR. Our findings imply that the absence of the BK_Ca_ channel might weaken the cellular response to DNA damage, potentially making cells more susceptible to PM-induced genomic instability. This not only enhances our understanding of environmental pollutant-induced genotoxicity but also paves the way for novel avenues of scientific inquiry into potential therapies targeting ion channels. However, our study primarily establishes a correlation rather than a direct causal mechanism. We assert that more targeted mechanistic studies are needed in the future, and we believe that the observed effects may be secondary to other cellular changes, such as changes in calcium homeostasis or levels of oxidative stress.

## Electronic supplementary material

Below is the link to the electronic supplementary material.

Supplementary Material 1

## Data Availability

All data generated or analyzed during the current study are included in the published article.
