# Supplementary material for: BKCa channel as a novel regulator of cellular DNA damage response in human bronchial epithelial cells in the presence of particulate matter
Source: Sci Rep. 2025 Jul 2;15:22789. doi: 10.1038/s41598-025-03824-9 (PMC12218102; doi:10.1038/s41598-025-03824-9)
Supplement: Supplementary file 1 — Supplementary Material 1 [file 41598_2025_3824_MOESM1_ESM.pdf]

Figure S1

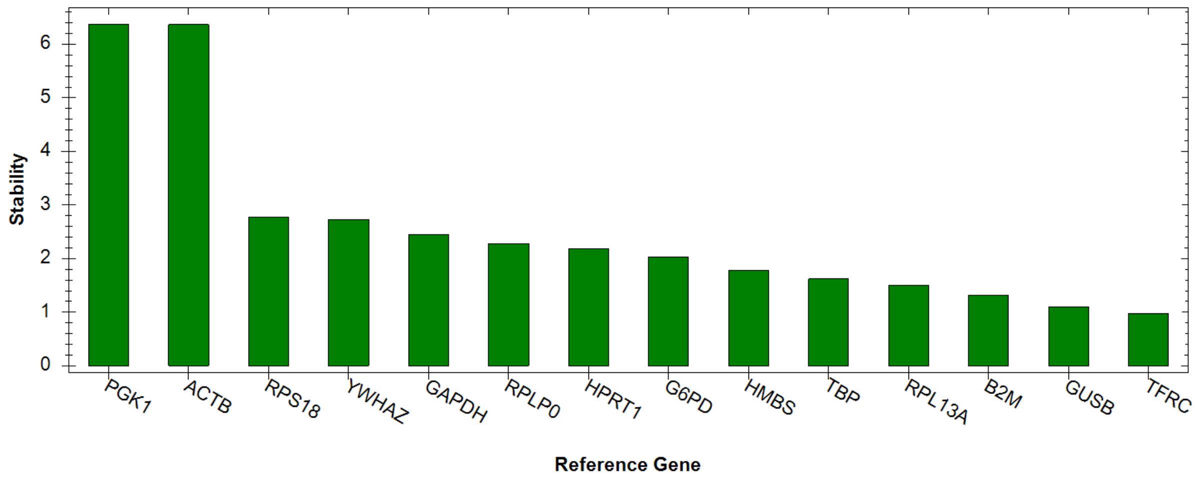

Figure S1. A graphical representation of candidate reference gene stability for HBE wt cells *vs.* HBE Δα BK$_{\mathbf{Ca}}$. The stability values are the log-transformed inverse of M values (Ln(1/AvgM) – the higher the bar, the more stable a gene is across the samples tested (n=3). The plot is color-coded as follows: ideal – green. The values were calculated automatically using the Reference Gene Selection Tool (Bio-Rad CFX Maestro software).

Figure S2

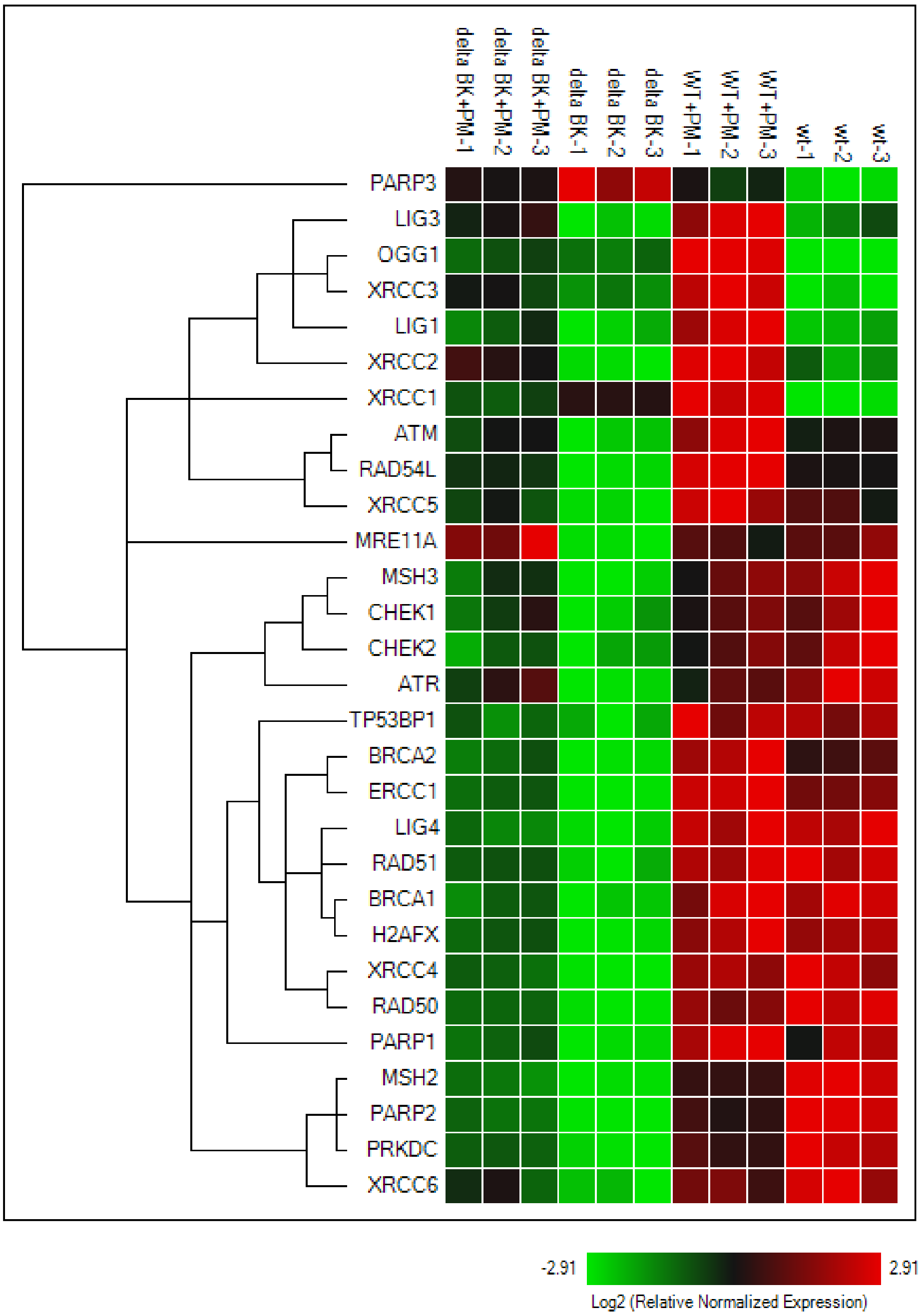

Figure S2. Combined cluster heatmap analysis of the normalized expression of DNA-damage signaling genes. Wt: HBE, wt + PM: HBE wt + PM, delta BK: Δα BK_{Ca}, delta BK + PM: Δα BK_{Ca} + PM. The red color characterizes a relatively high level of gene expression whereas the green color indicates a low level. The data were analyzed (n=3) and clustered by targets using the Reference Gene Selection Tool from Bio-Rad CFX Maestro software.

Figure S3

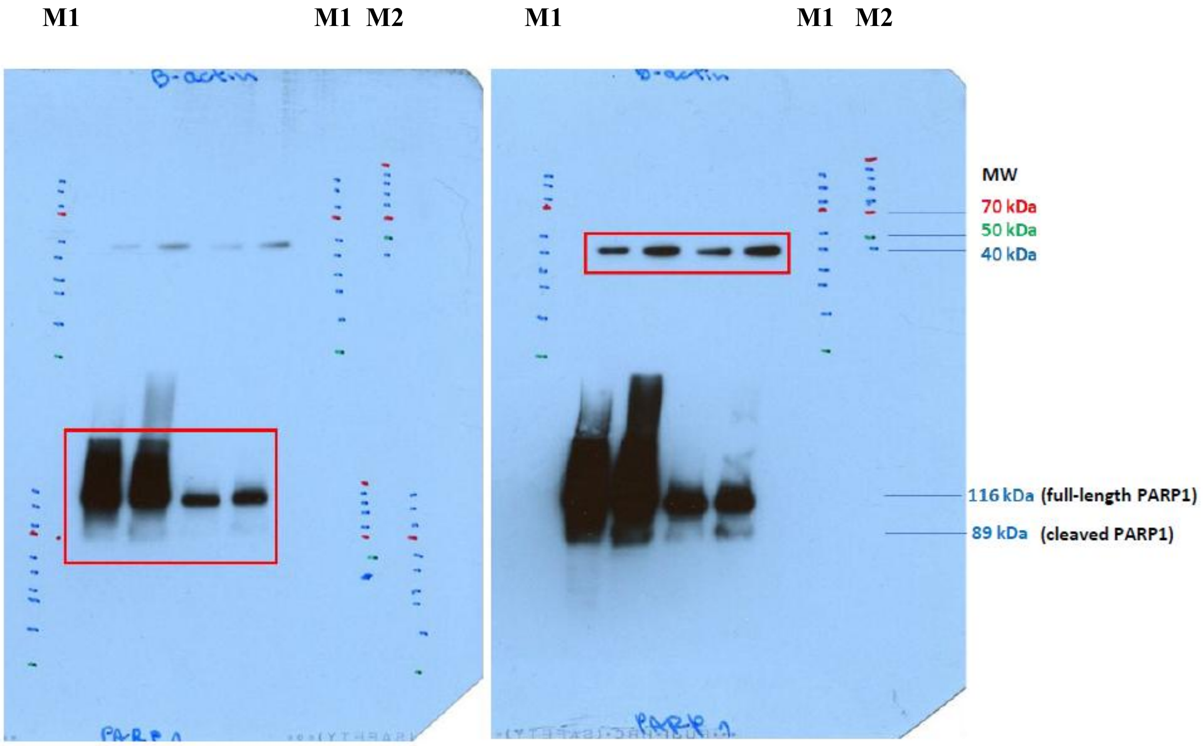

Figure S3. Images of original uncropped, unprocessed Western blots used for the preparation of Figure 2D and Figure 2E. 2 exposure times. Upper western blot bands: Anti-β actin; lower western blot bands: Anti-PARP Polyclonal Antibody, Thermo Fisher Scientific, USA, PA5-34803: full-length PARP1 – 116 kDa, cleaved PARP1 – 89 kDa. Samples are presented as follows from the left: 1: HBE wt, 2: HBE Δα BK_{Ca}, 3: HBE wt + PM, 4: HBE Δα BK_{Ca} + PM. The red boxes indicate the bands that were selected for publication. Protein ladders in the upper panel: M1: PageRuler™ Prestained Protein Ladder, 10 to 180 kDa (#26617, Thermofisher). M2: Spectra™ Multicolor High Range Protein Ladder (#26625, Thermofisher).

Supplementary Table S1.

The list of selected genes and their related DDR pathways.

Gene	DDR pathway
**ATM**	DNA double-strand breaks repair (DSBR)
**ATR**	DNA double-strand breaks repair (DSBR)
**BRCA1**	DNA double-strand breaks repair (DSBR)-HR
**BRCA2**	DNA double-strand breaks repair (DSBR)-HR
**CHEK1**	cell cycle arrest and DNA damage response
**CHEK2**	cell cycle checkpoint regulator
**ERCC1**	the nucleotide excision repair (NER)
**H2AFX**	DNA double-strand breaks repair (DSBR)
**LIG1**	the base excision repair (BER)
**LIG3**	single-strand breaks repair (SSBR)/BER
**LIG4**	DNA double-strand breaks repair (DSBR)-NHEJ
**MRE11A**	DNA double-strand breaks repair (DSBR)-NHEJ
**MSH2**	mismatch repair (MMR) pathway
**MSH3**	mismatch repair (MMR) pathway
**OGG1**	single-strand breaks repair (SSBR)/BER
**PARP1**	single-strand breaks repair (SSBR)/BER
**PARP2**	single-strand breaks repair (SSBR)/BER
**PARP3**	single and double-strand breaks repair (SSBR/DSBR)
**PRKDC**	DNA double-strand breaks repair (DSBR)-NHEJ
**RAD50**	DNA double-strand breaks repair (DSBR)-HR
**RAD51**	DNA double-strand breaks repair (DSBR)-HR
**RAD54L**	DNA double-strand breaks repair (DSBR)-HR
**TP53BP1**	DNA double-strand breaks repair (DSBR)-NHEJ
**XRCC1**	single-strand breaks repair (SSBR)/BER
**XRCC2**	DNA double-strand breaks repair (DSBR)-HR
**XRCC3**	DNA double-strand breaks repair (DSBR)-HR
**XRCC4**	DNA double-strand breaks repair (DSBR)-NHEJ
**XRCC5**	DNA double-strand breaks repair (DSBR)-NHEJ
**XRCC6**	DNA double-strand breaks repair (DSBR)-NHEJ
